# Targeting the dendritic cell-T cell axis to develop effective immunotherapies for glioblastoma

**DOI:** 10.3389/fimmu.2023.1261257

**Published:** 2023-10-20

**Authors:** Bryan Gardam, Tessa Gargett, Michael P. Brown, Lisa M. Ebert

**Affiliations:** ^1^ Adelaide Medical School, The University of Adelaide, Adelaide, SA, Australia; ^2^ Translational Oncology Laboratory, Centre for Cancer Biology, University of South Australia and South Australia (SA) Pathology, Adelaide, SA, Australia; ^3^ Cancer Clinical Trials Unit, Royal Adelaide Hospital, Adelaide, SA, Australia

**Keywords:** cancer, immunotherapies, dendritic cells, glioblastoma, T cells, combination therapies, CAR-T cells, brain tumor

## Abstract

Glioblastoma is an aggressive primary brain tumor that has seen few advances in treatments for over 20 years. In response to this desperate clinical need, multiple immunotherapy strategies are under development, including CAR-T cells, immune checkpoint inhibitors, oncolytic viruses and dendritic cell vaccines, although these approaches are yet to yield significant clinical benefit. Potential reasons for the lack of success so far include the immunosuppressive tumor microenvironment, the blood-brain barrier, and systemic changes to the immune system driven by both the tumor and its treatment. Furthermore, while T cells are essential effector cells for tumor control, dendritic cells play an equally important role in T cell activation, and emerging evidence suggests the dendritic cell compartment may be deeply compromised in glioblastoma patients. In this review, we describe the immunotherapy approaches currently under development for glioblastoma and the challenges faced, with a particular emphasis on the critical role of the dendritic cell-T cell axis. We suggest a number of strategies that could be used to boost dendritic cell number and function and propose that the use of these in combination with T cell-targeting strategies could lead to successful tumor control.

## 1 Introduction

Glioblastoma is the most common and aggressive form of malignant brain tumor (1). Between 2014 and 2018, the Central Brain Tumor Registry of the United States (CBTRUS) reported 133,973 glioblastoma cases; this represented 14.3% of all central nervous system (CNS) tumors. Of these patients, 94% were over the age of 40, while 5% were aged between 15 and 39, and the remaining 1% were less than 14 years old (2). Glioblastoma is considered a rare form of cancer with a diagnosis rate of less than 6 in 100,000 (3); however, the impact of this disease is profound as the prognosis of glioblastoma remains extremely poor, with patient survival being generally 7-17 months post-diagnosis (1, 4–6). In the 5 years covered in the CBTRUS report, glioblastoma patients had a 1-year overall survival rate of 40.9%, with this rate showing a rapid decrease to only 6.8% 5-year overall survival (2). There are few reports of costs in glioblastoma, and there is variance between countries and health care systems; nevertheless, the economic cost is substantial due to both direct healthcare costs and to the years of potential life lost to this disease (7).

Even with the many advances following single-cell sequencing and epigenetic profiling of glioblastoma tumors, there has not yet been translation from scientific discovery to new anti-cancer targets and drugs (8–10). With the exception of tumor treating fields (TTF) administered concurrently with standard post-surgical treatment, there has been no additional survival prolonging treatment in almost 20 years. Glioblastoma remains an incurable disease affecting a broad range of ages with devastating outcomes (1, 11, 12).

In this review, we look at the potential for immune based therapies to treat glioblastoma, and also the challenges facing these immunotherapies. In particular, we explore the benefits that may be achieved by targeting the dendritic cell-T cell axis.

## 2 Current standard treatment for glioblastoma

The Stupp protocol, developed almost 20 years ago, remains the standard treatment for newly diagnosed glioblastoma (11); this protocol involves surgery to remove as much of the tumor as is safely possible, followed by radiotherapy and chemotherapy with temozolomide (TMZ) (13). However, following this initial treatment regimen, tumor recurrence is almost inevitable. The importance of removing as much of the tumor as possible was quantified in a study by Stummer et al. that showed that total resection of the tumor was the only factor extending overall survival (14). These findings agreed with a previous study by Lacroix et al. that found that greater than 98% of tumor volume needed to be resected to increase overall survival (15). Even the addition of TMZ in the Stupp protocol only increased the average survival by approximately 2.5 months, and 84% of the patients had died by 2 years post-diagnosis (13).

**Figure 1:**
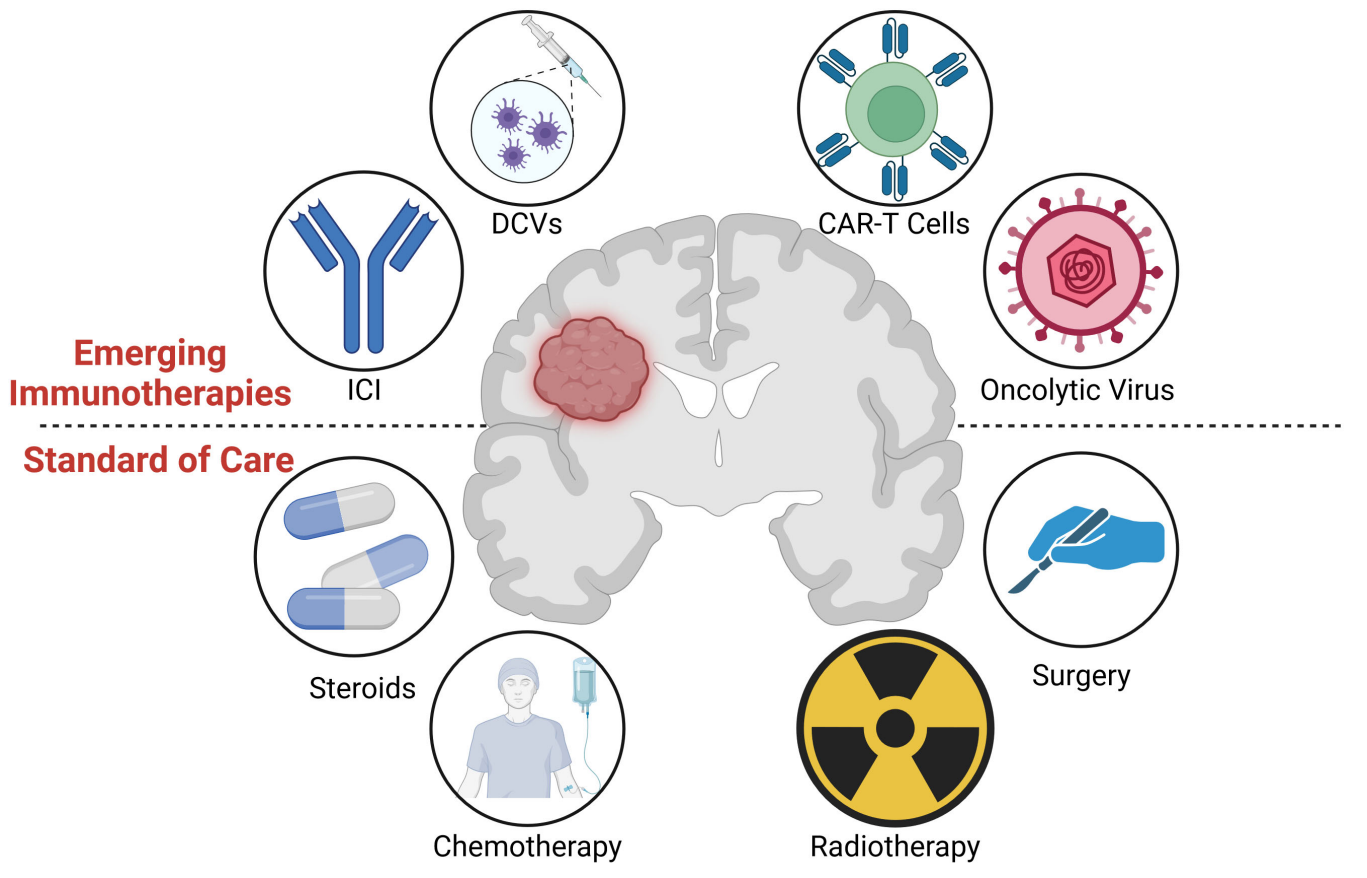
(Lower) Graphical overview of the standard of care treatment for glioblastoma, including surgery, radiotherapy, chemotherapy, and steroids. (Upper) Immunotherapies under investigation, including oncolytic viruses, CAR-T cells, dendritic cell vaccines (DCVs), and Immune Checkpoint Inhibitors (ICIs). Image created with Biorender.com.

No standard treatment regimen has been determined for elderly (>60-70 year old) glioblastoma patients who may not withstand the rigors of the Stupp protocol (16). Similarly, treatment of pediatric high grade gliomas is also considered palliative with survival rates of <10% beyond 2 years (17). Likewise, there is no standard second line survival prolonging treatment for recurrent glioblastoma, although bevacizumab may contribute to symptom control (18). Treatment options often depend on the individual and clinician, with survival generally less than six months following recurrence (13, 19, 20). Thus, new therapies, including immunotherapies, need to be developed for glioblastoma patients with the aim of extending the overall survival rates. An overview of the current standard treatments, as well as the immunotherapies discussed in section 5, is shown in **Figure 1**.

## 3 Anti-tumor immunity and dendritic cells

### 3.1 Anti-tumor immunity

From cancer initiation through to progression, interactions between cancer and immune cells proceed through three phases: elimination, equilibrium, and escape (21). Elimination is the phase where immune cells target and kill cancer cells; if successful, all cancerous cells would be removed from the body. However, any surviving cancer cells can persist and compete with the immune system in the equilibrium phase by continuous mutation and adaption. Finally, in the escape phase, the mutated cancer cells either become invisible or impervious to the immune system because of the advantages gained during the equilibrium phase (21), allowing them to grow and spread unopposed.

Immunotherapies aim to shift this balance in favor of the immune system to prevent cancer cell escape. These approaches are seen as a crucial new ‘fourth pillar’ of cancer treatment, in addition to the classical therapies of surgery, radiation and chemotherapy (22). Successful immunotherapy relies on the interaction of key immune cell subsets, including T cells and DCs. Therefore, an understanding of the interplay of these immune cells in the context of the tumor microenvironment is essential for advancing immunotherapy for glioblastoma.

### 3.2 Dendritic cells and their subsets

DCs are the most potent professional antigen presenting cells of the immune system. DCs integrate signals of the innate and adaptive components of the immune system and initiate adaptive immunity by activating T cells (23). Most cells of the body are able to express endogenous antigenic peptides on major histocompatibility molecule (MHC) class I molecules, which allows the identification of cancerous or virally infected cells by CD8^+^ T cells. In addition, all DC subsets have the ability to capture extracellular antigens and present processed peptides on MHC class II molecules to CD4^+^ T cells, while specialized subsets of DCs are also able to present extracellular antigenic peptides on MHC class I molecules to CD8^+^ T cells, a process called cross-presentation. Cross-presentation allows the priming of naïve CD8^+^ T cells specific to antigens external to the DCs, which is crucial to initiating the cytotoxic T cell responses required for anti-tumor immunity (24). Together with antigen presentation, naive T cells require set co-stimulatory signals for activation. DCs provide this additional signal through CD80 or CD86, which are upregulated on the DC surface after antigen capture (25). DCs are thus a critical immune cell type in clearing the body of cancerous cells.

DCs in the tumor microenvironment (TME) are important for the immune system’s anti-tumor effects but can also support tumor growth when present in a tolerogenic form (26). The multiple roles played by DCs in the anti-tumor or pro-tumor response depends on the DC subsets present in the TME. Although only limited data are available on the function of some DC subsets in glioblastoma, other studies in solid tumors may inform the likely DC functions in glioblastoma, and therefore these are also reviewed below. The issue of DC heterogeneity was identified in a detailed review by Pombo Antunes et al. which identified that the DCs characterized in other solid tumors may not translate to glioblastoma and highlighted the need for further analysis of glioblastoma DC subsets (27).

#### 3.2.1 Plasmacytoid dendritic cells

pDCs in humans are identified as human leukocyte antigen-DR (HLA-DR)^+^ CD123^+^ CD304^+^ CD303^+^ (28). pDCs produce high levels of type 1 interferons (IFN) and play an essential role in viral clearance (29). Further, it is now accepted that mature pDCs can present antigenic peptides and activate naïve CD4^+^ T cells (30), although the ability of pDCs to cross-present antigens to CD8^+^ T cells remains unresolved (31). In cancer, pDCs have been reported to induce regulatory T cells (T_reg_) cells in solid tumors, and have been associated with poor outcomes (32, 33). A study of ovarian cancer patients found a negative correlation between the number of infiltrating pDCs and patient outcomes (32). In keeping with this observation, a study in a humanized murine xenograft model of human melanoma found that pDCs were linked to the generation of T helper Type 2 (Th_2_) CD4^+^ T cells, which may promote rather than control tumor growth (33). Conversely, in an immunocompetent murine lymphoma model, pDCs were reported as potent activators of T Helper Type 17 (Th_17_) cells and cytotoxic T cells leading to an effective anti-tumor response (34). A review by Macri et al. reported that both Th_17_ and Th_2_ responses were observed after activation of pDCs (23). A pre-clinical study in a rat model of glioblastoma has identified the recruitment and activation of pDCs; however, the study also concluded that the role of pDCs in an anti-tumor response remained unclear (35).

Thus, the complicated roles of pDCs remain to be fully explored, and it remains unknown whether pDCs support an anti-tumor response or contribute to glioblastoma tumor progression.

#### 3.2.2 Conventional dendritic cells

Type 1 cDCs (cDC1s) are potent as cytotoxic T cell activators and efficiently present and cross-present antigens on MHC II and MHC I, respectively. In humans, cDC1s are typically identified as HLA-DR^+^ CD11c^+^ CD123^-^ CD11b^-^ CD141^+^ C-type lectin domain family 9 member A (Clec9A)^+^ (23). cDC1s are excellent antigen cross-presenting cells and lead to high levels of cytotoxic T cell activation in response to tumors (36, 37). Numerous pre-clinical tumor models have identified a role for cDC1 in T cell recruitment to the tumor and in supporting T cell-mediated and NK cell-mediated tumor control (38–40). Further, cDC1s have been identified in studies of murine fibrosarcoma models to be required for successful anti-tumor responses, including the recruitment of effector cells including T cells (41). A study in mice with orthotopic glioblastoma tumors by Bowman-Kirigin et al. demonstrated that cDC1s cross-presented antigens and were required for the priming of CD8^+^ T cells in the brain and draining lymph nodes, in keeping with other solid tumor studies (42). Likewise, Friedrich et al. found, also in a mouse orthotopic glioblastoma model, that antigen cross-presenting DCs were crucial for the activation of CD8^+^ T cells; however, they also identified differences in the DC numbers and phenotype depending on whether or not the isocitrate dehydrogenase (IDH) gene was mutated in the tumor cells (43).

Type 2 cDCs (cDC2s) in humans are identified as HLA-DR^+^ CD11c^+^ CD141^+^ CD1^low/+^ Clec10A^+/-^ CD5^+/-^ (28). Further subdivision of the cDC2 population into CD5^+^ and CD5^-^ subsets has also recently been reported (28). The CD5^+^ subset of cDC2 cells was found to have a greater ability to migrate to sites of infection compared to the CD5^-^ subset; however, they produced a suppressive T cell phenotype, whereas CD5^-^ cells elicited a stronger cytotoxic T cell effect (44). A recent study identified cDC2s as the most abundant DC subset in glioblastoma patient tumors, with the majority of these cells being CD5^-^ (26). Although it is widely believed that cDC1s are pivotal for anti-tumor responses, there is an argument for further investigation of CD5^-^ cDC2s in glioblastoma, as the significance of these cells may be under appreciated.

#### 3.2.3 Monocyte-derived dendritic cells and DC3

moDCs are viewed as a major subset of DCs of heterogeneous composition that can be generated easily *in vitro* (45). moDCs are also found *in vivo*, in both humans, and murine models (46, 47). moDCs in humans are reported as HLA-DR^+^ CD1c^+^ CD14^+^ CD226^+^ CD163^-^ (28). Conversely, DC3s are a relatively newly defined subset of DCs that resemble moDCs and are thought to derive from moDCs (28, 48). DC3s in humans are identified as HLA-DR^+^ CD1c^+^ CD14^+^ CD163^+^ CD5^-^ (23, 49). However, a recent study by Bourdely et al. defined DC3s as a discrete subset unrelated to moDCs and found them to be prevalent in solid tumors, where they promote the generation of resident memory T cells (T_RM_) (49). Ex vivo generation of both moDCs and DC3s can be achieved using the combination of granulocyte-macrophage colony-stimulating factor (GM-CSF) and interleukin 4 (IL-4) (49–53), suggesting these populations share similarities. DC3 cells have been detected in samples of both lung and breast cancer (54, 55). Although there was no correlation between DC3s and patient outcomes in lung cancer, a positive correlation was observed in triple-negative breast cancer patients (55). Additionally, DC3s have been reported in glioblastoma by single-cell RNA sequencing (scRNAseq) (56). However, data confirming the presence of DC3s using protein markers is yet to be reported.

The true origin of moDCs remains contentious, and whether indeed they are related to cDC2 and DC3 cells is an area identified for continued investigation (28, 48, 57). For the purpose of this review, we will not distinguish between moDCs and DC3s.

## 4 Barriers to anti-tumor immunity in glioblastoma

### 4.1 The Blood-brain barrier

The BBB consists of specialized vessels with tight junctions in the CNS that control the transfer of cells and molecules between the blood and the CNS (58) while also preventing toxins and disease- causing agents from entering the CNS (59). When a tumor encroaches on the BBB, it forms a more permeable blood-tumor barrier (BTB) that can permit the entry of small and larger molecules into the main tumor mass. However, the BTB remains heterogeneous, and the passage of molecules and immune cells depends on the location and type of cancer (60). Moreover, the BBB remains a significant obstacle to the efficient delivery of therapeutic agents to the tumor-adjacent normal brain tissue, where invading tumor cells spread, and thus limits the effectiveness of some cytotoxic drug and antibody therapies (61, 62).

The CNS was long considered to be an immune privileged site (63), and the BBB was accordingly thought to limit treatment of glioblastoma with immunotherapies (61). Now, however, evidence that CNS antigens drain through lymphatic vessels to the deep cervical lymph nodes (64, 65) indicates that immune responses can be initiated from antigens within the brain. This highlights the key role that DCs can play in initiating and maintaining T cell responses to brain tumors.

### 4.2 DCs exist among the immunosuppressive tumor microenvironment of glioblastoma

The paucity of DCs in brain tumors and their functional impairment is likely an intrinsic feature of the immunosuppressive TME of glioblastoma, which has been recognized since the early 1970’s (66) and induced by numerous mechanisms, which have been reviewed recently and comprehensively by others (67). Briefly, the glioblastoma TME consists of many different immune cell types, including T regulatory cells (T_reg_), tumor-associated macrophages (TAMs) and suppressive myeloid cells (68). Remarkably, myeloid cells can make up to 50% of the glioblastoma tumor mass (20), and Friebel et al. reported that up to 80% of all leukocytes in the TME consisted of TAMs and monocytes (9) They have thus been a major focus of investigation (69, 70). The tumor and the TME secrete molecules and cytokines that polarize the TAMs to an immunosuppressive phenotype (71). Further, TAMs can then inhibit T cell functionality via production of immune-suppressive factors such as IL-10 (72). There is increasing evidence of substantial heterogeneity in the glioblastoma TME between patients, including the presence of cDC1s, cDC2s, and pDCs (70, 73), and changes in cell populations have been seen at different stages during a patient’s disease in cDC1s, and pDCs (74).

In addition, recent studies argue that the TME in the brain prevents effective antigen presentation by myeloid lineage cells, including cDC1s, cDC2, and pDCs, and that this is a more important factor leading to ineffective anti-tumor responses than the overall suppressive nature of the TME (75, 76). While the exact mechanism is not fully understood, Simonds et al. reported in a glioblastoma murine model, response to immune checkpoint inhibitors (ICI) relied on T cells and DCs, and mice with subcutaneous tumors could respond to ICI while mice with intracranial tumors could not, indicating the location of the tumor in the brain was a critical factor influencing immune recognition (75). It should be considered that the TME, whether by preventing antigen presentation or its directly suppressive nature, is a significant barrier to treating glioblastoma patients with immunotherapies. There is still extensive work to be done in characterizing the complexity of the immune microenvironment associated with glioblastoma, including a characterization of rarer T cell and DC subsets.

### 4.3 The lymphopenia of glioblastoma and implications for developing DC-based therapies

The immune suppression associated with glioblastoma extends beyond the TME and is manifest globally as lymphopenia. The origins of lymphopenia in glioblastoma patients are related both to the disease itself and its treatment. The number of circulating lymphocytes is homeostatically regulated and may reduce with age, or from specific diseases and their treatments (77). Therefore, these effects may have significant implications for the development new immunotherapies for glioblastoma, including those that target the DC-T cell axis.

Chongsathidkiet et al. made the striking observation that lymphopenia resulted in sequestration of T cells in the bone marrow when a brain tumor was also present. A possible mechanism is tumor-mediated downregulation of sphingosine-1 phosphate receptor-1 (S1PR1) on T cells restricting T cells egress from bone marrow (78). Interestingly, this finding was not only found in primary brain tumors but also in other tumors that were xenografted into the brains of mice. This result points to a possible hypothesis that it is an effect specific to the brain, deriving from either from the tumor or TME development, which may be self-protective and prevent inflammation by systemic effects on cells of the immune system.

We found that clinical use of cytotoxic chemotherapy drugs can be associated with an increase in the number of effector cells in recovery phase and that repopulation of the lymphocyte compartment can be associated with a concomitant reduction in suppressive phenotypes in a drug dependent way (79). In glioblastoma patients, lymphopenia often follows treatment with TMZ, especially when combined with radiotherapy, and is associated with a reduction in survival (80). In contrast, another study reports treatment benefits following TMZ treatment-induced lymphopenia (81). A recent study by Ghosh et al. showed that lymphopenia was induced in glioblastoma patients following radiation therapy, which correlated with a reduction in progression-free and overall survival (82). Interestingly, these effects were driven by increased myelopoiesis and the generation of myeloid-derived suppressor cells (MDSC), which in turn were associated with T-cell lymphopenia.

### 4.4 Loss of DC function

It has been reported that DCs are lacking in the TME of patients’ glioblastoma tumors (83, 84). However, a recent study by Simonds et al. has shown that DCs are still present in human glioblastoma, although reduced compared to other tumor locations. This study also highlighted that a deficiency in DCs can lead to a delayed immune response through poor priming of T cells in the draining lymph nodes (75). This finding supports a significant role for DCs in immune responses to tumors, including glioblastoma. In addition to the reduced numbers, the functionality of DCs has also been reported to be impaired in glioblastoma patients compared to healthy patient samples (26, 43, 56, 83–89). A summary of the reported changes in DC numbers in glioblastoma patients is presented in **Table 1**, including analyses of circulating DCs as well as those within tumors. Together, these studies suggest that reduced quantity and quality of DCs in glioblastoma may impair tumor control and the effectiveness of immunotherapies. Although other studies have identified DCs in the tumor and TME of glioblastoma patients from tumor samples (42) or scRNAseq data (43, 56, 91), no quantification compared to healthy patients was reported in these studies.

**Table 1:** Changes in dendritic cells in glioblastoma patients.

DC Cells Identified	Markers Used	Sample	Observation	Detection Method	Ref
cDC1s	CD19^-^ CD14^-^ HLA-DR^+^ CD141^+^	Peripheral Blood (Fresh whole blood)	cDC1s – Reduced by 40% ^1^	Flow Cytometry	(88)
cDC2s	CD19^-^ CD14^-^ HLA-DR^+^ CD11c^+^		cDC2s – Reduced by 65% ^1^		
pDCs	CD19^-^ CD14^-^ HLA-DR^+^ CD303^+^		pDCs – Reduced by 60% ^1^		
Total DCs	(cDC1) XCR1^+^ CLEC9A^+^ CADM1^+^	Fresh Tumor Samples	Primary glioblastoma <2% of CD45^+^ cells^2^	scRNA seq	(26)
	(cDC2) FceR1A^+^ CLEC10A^+^ CD1C^+^		Recurrent glioblastoma 2-8% of CD45^+^ cells^2^		
	(pDC) IL3RA^+^ LILRA4^+^				
Total DCs	(DCs) HLA-DR^+^ Lin^-(3)^ CD45^+^ CD11c^+^	Peripheral Blood (Fresh whole blood)	Total DCs were reduced by 84%^4^	Flow Cytometry	(83)
pDCs	(pDC) HLA-DR^+^ Lin^-(3)^ CD45^+^ CD123^+^		pDCs were reduced by 78%^3^		
cDC2s	(cDCs) SSC^low^ CD1c^+^	Peripheral Blood (Fresh whole blood)	cDC2s – reduced by 55% pre-operative^1^	Flow Cytometry	(84)
			cDC2s – reduced by 32% post-operative^1^		
pDC2s	(pDCs) SSC^low^ CD303^+^		pDCs – reduced by 68% pre-operative^1^		
			pDCs – reduced by 80% post-operative^1^		
Total DCs	HLA-DR^+^ CD45^+^ CD11b^+^ CD11c^+^	Cerebrospinal Fluid	50% increase in DCs^1,5^	Flow Cytometry	(85)
cDC2s	(DC2s) Lin^-(6)^ HLA-DR^+^ CD11c^+^	DCs purified from peripheral blood mononuclear cells	cDC2 – reduced by 22%^1^	Flow Cytometry	(86)
pDCs	(pDCs) Lin^-(6)^ HLA-DR^+^ CD123^+^		pDC – reduced by 15%^1^		
cDC1s	(cDC1s) Lin^-(7)^ HLA-DR^+^ CD11c^+^ CD1c^+^	Peripheral Blood (Fresh whole blood)	No DCs were identified in glioblastoma Patients	Flow Cytometry	(87)
cDC2s	(cDC2s) Lin^-(7)^ HLA-DR^+^ CD11c^+^ Clec9A^+^				
pDCs	(pDCs) Lin^-(7)^ HLA-DR^+^ CD11c^-^ CD123^+^				

1. Compared to healthy controls, 2. cDC2s were identified as the most abundant, 3. Lin = CD3 CD4 CD25 CD34 4. Compared to healthy results by Autissier et al. (90) expressed as % of total white blood cells, 5. Reduced activation markers HLA-DR, CD40, CD86, 6. Lin = CD3 CD14 CD19 CD20 CD56 CD34. 7. Lin = CD3, CD14, CD19, CD20, CD56.

Adhikaree et al. reported downregulation of HLA-DR, the T cell costimulatory molecule CD86 and increases programmed death-ligand 1 (PD-L1) when DCs were exposed to glioblastoma tumors or the corticosteroid dexamethasone, which is often given to glioblastoma patients to reduce brain swelling and relieve symptoms (88). This phenomenon has also been reported elsewhere (92). The downregulation of HLA-DR and CD86 indicates a less mature DC phenotype and can lead to the generation of T_reg_ cells (88); thus, different approaches have been used to restore the function of the DCs, including inhibiting p38 (88) and through silencing PD-L1 in DCs (92). Although no studies have reported other effects of p38 inhibition, p38 is responsible for the regulation of many transcription factors, including pro-inflammatory cytokines, growth factors, and adhesion molecules (93), so further studies are required to investigate the biologic specificity of p38 inhibition.

**Figure 2:**
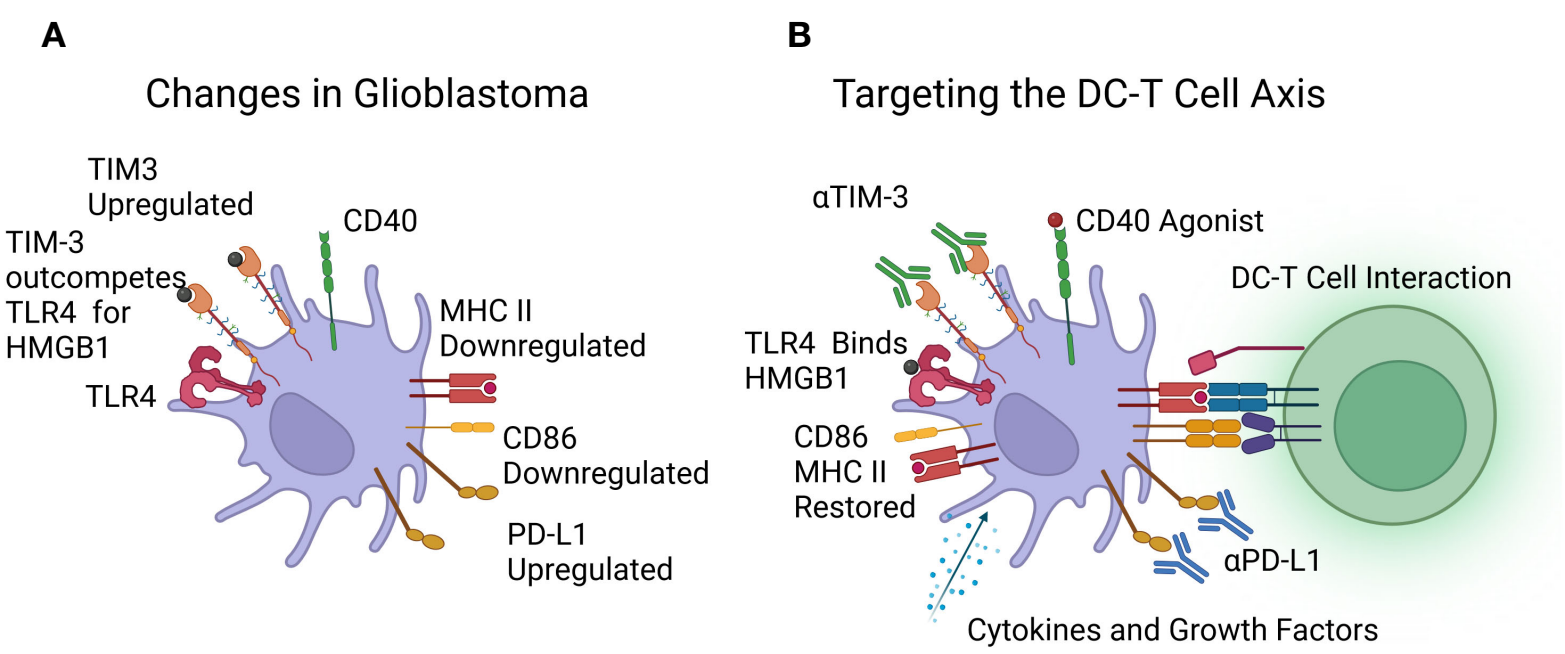
**(A)** Reported changes in dendritic cells in patients with glioblastoma, including downregulation of MHC class II and CD86, upregulation of PD-L1 and TIM-3, with TLR4 outcompeted. **(B)** Immunotherapies under investigation to target the DC-T cell axis, including cytokines, growth factors, and CD40 agonists to boost dendritic cell numbers and function, and Immune Checkpoint Inhibitors (ICIs) targeting TIM-3 and PD-L1. Image created with Biorender.com.

A graphic representation of the changes seen in dendritic cells is shown in **Figure 2A**.

## 5 Targeting the DC-T cell axis with immunotherapies

### 5.1 Chimeric antigen receptor T cells and DCs

CAR-T cell therapy generally involves genetically modifying autologous T cells to express high-affinity chimeric receptors, with the extracellular region specific for tumor antigens and the intracellular portion responsible for T cell activation upon CAR engagement. This receptor facilitates tumor antigen recognition by the engineered T cells and leads to efficient tumor cell killing (94–96). CAR-T cell treatment involves the collection of the patient’s T cells, gene modification for CAR expression, CAR-T cell expansion, and patient infusion with the CAR-T cell product. The first generation of CAR-T cells entered clinical trials in the 1990s’ as a treatment for HIV (97). Second- and third-generation CAR-T cells were developed to include one or multiple costimulatory domains, respectively (98). CAR-T cells have been highly effective in treating B-cell malignancies (99–101); however, the same cannot be said for solid tumors (95). Currently, CAR-T cells targeting multiple antigens are being investigated to treat glioblastoma in preclinical studies and early clinical trials (102, 103). To address the heterogeneity of glioblastoma, multi-antigen targeting by CAR-T cells is being explored in some of these studies (104), but tumor specificity is required to minimize off-tumor, on-target toxicity in normal healthy tissue (105). Notwithstanding the relatively early stage of development of CAR-T cell therapy for glioblastoma, limited effectiveness or resistance to treatment has already been reported, including loss of tumor antigen expression (11, 106) and presumed CAR-T cell induced resistance through the upregulation of programmed death-ligand 1 (PD-L1) and recruitment of T_reg_ cells (107). The likely reasons given for these modest clinical results include the immunosuppressive nature of the TME (96, 108), the high degree of antigen heterogeneity (109), sub-optimal CAR-T cell doses, and route of administration (103).

To address some of the challenges associated with treating solid tumors, including glioblastoma, recent CAR-T cell designs may incorporate additional genes to improve T cell function. For example, cytokine-enabled CAR-T cells, which are sometimes referred to as TRUCKs (T cells redirected for antigen-unrestricted cytokine-initiated killing), secrete T cell supportive cytokines or cytotoxic mediators such as IL-12, IL-15 and IL-18 (110). However, the ability of local, CAR-T-produced cytokines to promote beneficial paracrine changes in the immune microenvironment, including DC stimulation, has also been explored. For example, IL-18-producing TRUCKs reportedly induce changes in the balance of M1 and M2 macrophages and may also alter the phenotype of from suppressive cDC2s, although a change in cDC1s numbers was not observed (111). IL-12 producing CAR-T cells are perhaps the most widely studied TRUCKs to date (112–114); however, there are limited studies of the effects of IL-12 on the TME or DCs. There has been one study of IL-12 enhanced CAR-T cells in an immune-competent murine lymphoma model, which reported evidence of epitope-spreading, implying DC involvement, and another study describing the effect of murine CAR-T cells co-administered with local IL-12 delivery in the murine GL261 glioma model, which has confirmed that in principle IL-12 can re-shape the myeloid compartment in solid tumors (115). Our recent studies of GD2-CAR-T cells expressing transgenic IL-15 also suggest beneficial effects on DCs (116).

While the approaches above may indirectly act on DCs, a promising approach to overcome DC deficits in glioblastoma is to directly enhance intra-tumoral DC number and function via using TRUCKs as a delivery vehicle for DC-stimulating agents. Of particular note, a recent preclinical study by Swan et al. investigated CAR-T cells expressing mouse IL7 and FMS-like tyrosine kinase 3 ligand (Flt3L) in a glioblastoma mouse model. These CAR-T cells increased the number of antigen-presenting cDC1s and increased CAR-T cell survival; further, it was noted that this combination improved the overall survival of the mice (117). Similar results, including an increased number of cDC1s in the tumor, were observed by Lai et al. in other non-glioblastoma cancer models (118). Furthermore, Kuhn et al. found in a mouse B cell lymphoma model that CAR-T cells expressing CD40L stimulated DC1s to activate endogenous T cells and provide greater anti-tumor effects (119). It will be very interesting in future studies to investigate whether similar effects are observed in the context of glioblastoma.

### 5.2 Dendritic cell vaccines

DCV approaches involve the ex vivo culture and maturation of autologous DCs with antigens derived from tumor lysate or specific peptides before the patient is infused with the preparation to activate a T-cell response (120). A comparison of the route of administration was conducted in patients with advanced melanoma and found that intradermal injection resulted in 4% of the DCs migrating to the draining lymph nodes. In contrast, intranodal injection resulted in up to 84% of the DCs migrating to adjacent lymph nodes (121). Like CAR-T cell therapies, the DCV process is personalized and expensive (107). Tumor lysates enable multiple antigens to be loaded onto the DCs; alternatively, specific peptides may be loaded onto the DCs. Most DCV trials use moDCs generated from peripheral blood mononuclear cells (PBMCs) with GM-CSF and IL-4, although the way the DCs are matured prior to infusion differ from one study to the next. A detailed comparison of five different DC phenotype maturation processes used in DCV clinical trials was conducted by Zhang et al., who demonstrated that the protocol utilizing four cytokines produced a more mature DC product (122).

Numerous glioblastoma clinical trials have been conducted, with mixed results, and not all living up to the expectations generated by preclinical results (51, 84, 123–126). For example, a clinical trial by Prins et al. compared tumor lysate and specific peptides as a source of antigens and found dose-limiting toxicity, but no apparent benefit for either approach and no overall patient benefit (125). In the DCV phase II randomized clinical trial in glioblastoma patients by Buchroithner et al., the tumor lysate approach was used and showed no overall increase in patient survival (126). Similarly in a randomized double-blind placebo-controlled phase II DCV trial with six peptides by Wen et al. there was no overall survival benefit, although a prolonged progression-free survival was noted in the treatment group (51). On the other hand, a clinical trial by Wang et al. found that treatment with a tumour-associated antigen (TAA) based personalized DCV showed treatment benefits warranting further exploration in a larger cohort (127).

An important point to note is that the DCV approach relies on the patient having an intact functional immune system. However, as noted earlier, glioblastoma has deleterious effects on circulating T cells and DCs and may impair the effectiveness of these therapies. However, targeting the DC – T cell axis in combination may overcome these challenges.

### 5.3 Oncolytic viruses may be a vehicle for stimulating DC number and function

OV therapy uses viruses that are only weakly pathogenic and have been genetically engineered to maximize anti-cancer effects while minimizing damage to normal cells. Additionally, some OV therapies have further genetic modifications to express immunomodulatory factors (128). Three of the main viruses being studied in glioblastoma include herpes simplex virus (HSV), adenovirus (Adv), and poliovirus; however, the effectiveness of OV in treating glioblastoma has been limited to date despite promising preclinical results (129). Although some of the challenges involve overcoming the suppressive TME, it is suggested that the OV generates its own immune response, and this response neutralizes the virus particles, thus reducing the overall effect (128).

To improve the efficacy of OV therapy, some investigators are engineering viruses to express Flt3L to enhance intra-tumoral DC numbers. For example, King et al. established a rat intracranial glioblastoma model in which Flt3L and herpes simplex virus type 1–thymidine kinase, delivered intratumorally by replication-defective recombinant adenovirus type 5 vectors, was able to control tumors in combination with the antiviral drug Ganciclovir. It was proposed that this was through the recruitment of pDCs (130). However, FLT3L has also been shown to expand cDCs (118, 131). In a similar study, Ali et al. showed a similar response in the same model with Flt3L alone (132). However, it should be noted that in the study by Ali et al., the treatment was administered three days post-cell implantation, which may lack clinical relevance as the tumor may not have established.

### 5.4 Immune checkpoint inhibitors and DC stimulation

Immune checkpoint inhibitors target immune checkpoint pathways with antibodies that disrupt the receptor/ligand interactions to prevent tolerance and restore the function of immune effector cells (133). Programmed cell death protein 1 (PD-1) with its ligand PD-L1, provides an inhibitory mechanism in activated T cells, B cells, monocytes and DCs to prevent the recognition of self-antigens and autoimmunity (134). Many cancers, including glioblastoma, upregulate PD-L1 on DCs, causing tolerogenic cell phenotypes to develop and, in turn, tumor escape (104). Similarly, cytotoxic T-lymphocyte–associated antigen 4 (CTLA-4) is expressed on T cells and, when engaged by its ligands CD80 or CD86, also suppresses T cell function (104).

Antonios et al. found increased expression of PD-L1 in glioblastoma tumor-infiltrating myeloid cells, and greater efficiency of a DCV was achieved in a preclinical murine glioblastoma model when combined with a PD-1 blocking monoclonal antibody (135, 136). A further study by Aslan et al. in a murine glioblastoma model identified the importance of PD-1, PD-L1 and CTLA-4 in T cell effector function while also acknowledging that other suppressive molecules may be at play on TAMs in the TME (137). In comparison, two reports from the clinical trials of anti-PD-1 agent nivolumab (CheckMate 143) were reported by Omuro et al. in newly diagnosed and recurrent glioblastoma patients. Both reports found that monotherapy treatment was safe, as was combination with CTLA-4 inhibition or standard-of-care treatments; however, there was no increased overall survival. In both cases, it was concluded that further studies and trials were warranted to identify approaches to use these novel agents more effectively, including in combination with other therapies (138, 139).

Although many combination experiments in the setting of glioblastoma are yet to be conducted, findings from other tumor models provide support for novel therapies that enhance DC function while also blocking immune checkpoint function. For example, Salmon et al. showed that treatment with Flt3L resulted in more DCs at the tumor site in a mouse melanoma model. However, DC1s in tumor-draining lymph nodes of these mice upregulated PD-L1, suggesting that a combination of Flt3L and anti-PD-L1 would result in greater T cell activation (131). Indeed, in a separate study, the same approach to expand DCs led to better outcomes following anti-PD-1 therapy in a melanoma mouse model (140).

T cell immunoglobulin and mucin domain-containing protein 3 (TIM-3) is another candidate for ICI therapy, although not yet tested clinically in glioblastoma. In addition to its function as a T cell checkpoint receptor, TIM-3 also regulates DC function. In this context, TIM-3 competes as a receptor for the nuclear alarmin protein high-mobility-group box 1 (HMGB1) secreted by dying tumor cells. HMGB1 normally functions as a danger signal, resulting in DC activation following binding to toll-like receptor 4 (TLR4) (141). However, Chiba et al. identified secreted factors in the TME that result in increased expression of TIM-3 on DCs (142). The increased expression of TIM-3 outcompetes TLR4, thus resulting in reduced activation of DCs and, in turn, reduced immune responses. Similarly, another ligand of TIM-3, galectin 9 has been recently identified as upregulated in the TME of melanoma patients (143) and glioblastoma patients (85). The increased presence of galectin 9 in the TME is also associated with a decreased ability of DCs to present tumor antigens.

Several additional studies support the critical role that DCs play in mediating responses to ICI agents. For example, in a subcutaneous mouse tumor model, Peng et al. identified that functional DCs were vital to an effective outcome from anti PD-L1 therapies (144). Similarly, a study in non-small cell lung carcinoma by Cohen et al. found that following PD-1 blockade, DCs are crucial for the reactivation of T cells (145). A recent study by Tomaszewski et al. found in glioblastoma that the upregulation of calmodulin-dependent kinase kinase 2 (CaMKK2) reduced the interaction of DCs with T cells, and reduced the effectiveness of ICI therapies; it was thus suggested that therapies targeting CaMKK2 and DC or T cells would enhance treatment outcomes, although there are no reports of these combination therapies being tested.

In support of these mechanistic studies, a number of reports confirm that combining ICI agents with DC-stimulating agents can promote anti-tumor activity in various cancer types. For example, preclinical mouse breast and pancreatic cancer models demonstrated that ICI therapy combined with a CD40 agonist increased immune cell infiltration and greater tumor control than ICI therapy alone (146). Likewise, in a further preclinical mouse model of pancreatic cancer, ICI therapy and CD40 agonists also showed increased tumor control (147), and an early clinical trial in metastatic colorectal cancer and metastatic pancreatic cancer showed that combining ICI therapy with a toll-like receptor 9 (TLR9) agonist also resulted in an increase in T-cell and DC tumor infiltration (148).

Considering the deficiencies in DC number and function in glioblastoma patients (see above and **Table 1**), these studies in other tumor types provide a strong rationale to test approaches to support DC maturation and function to improve the efficacy of ICI therapies in treating glioblastoma.

The possible improved outcomes by targeting the DCs with immunotherapies and targeting the DC-T cell axis is shown at **Figure 2B**.

## 6 Glioblastoma immunotherapy clinical trials

Despite positive preclinical data, many of the single CAR-T cell, DCV and OV trials have had limited success in treating glioblastoma, and these reports often note that better success may be seen when combined with checkpoint inhibitors or other approaches. This further highlights the need for a holistic approach to immunotherapies for the treatment of glioblastoma; further, given the heterogeneity of glioblastoma tumors, and the immunosuppressive mechanisms operating in the TME, combined immunotherapies are more likely to be effective. However, according to the U.S. National Library of Medicine clinical trials database (https://www.clinicaltrials.gov/) (149), a search for active, recruiting, and not yet recruiting clinical trials testing immunotherapies for glioblastoma returned a total of 43 trials, but of these, only 11 involved a combination of 2 immunotherapies 9 of which are a combination of 2 checkpoint inhibitors, and just 1 trial consisted of a combination of 3 immunotherapies (**Table 2**). Of particular note, only 1 trial is testing combinations of DC-targeted approaches with any other type of immunotherapy. This is clearly an area that deserves further attention.

**Table 2:** Current clinical trials for glioblastoma with Immunotherapies registered on the U.S. National Library of Medicine clinical trials database.

Immunotherapy Treatment	Targets^1^	Disease Stage^2^	Trial Phase	Trial Status^3^	Trial number
Dendritic Cell Vaccine	Th-1 DCVax-L Tumour Lysate WT1 mRNA CD200AR-L and GBM6-AD Tumour Lysate pp65-flLAMP, GM-CSF GSC-DCV and αPD-1 Tumour Lysate and IL-12	ND ND ND ND R ND ND R TN	1 3 1,2 1,2 1 2 1 2 1,2	R ANR R R ANR R ANR R NYR	NCT04552886 NCT00045968 NCT04801147 NCT02649582 NCT04642937 NCT03395587 NCT04963413 NCT04888611 NCT04388033
CAR-T Cells	CD44 and CD133 CD70 UNK NKG2D	R ND R ST	1 1 1 1	NYR R R R	NCT05577091 NCT05353530 NCT05660369 NCT05131763
γδ T Cella	UNK UNK	ND, R ND	1,2 1	R R	NCT05664243 NCT04165941
Immune Adjuvants	GM-CSF, Sapylin, MnCI2 CD38	R ND	1,2 1,2	R R	NCT05131711 NCT04922723
Gene Therapy	IGF1R WT1, PSMA, hTERT, IL-12	ND ND	2 1,2	R ANR	NCT04485949 NCT03491683
Checkpoint Inhibitors	PD-1 TIM-3 and PD-1 GITR and PD-11 PD-1 and TIGIT PD-1 mTORC1 and BTK CTLA-4 and PD-1 CTLA-4 and PD-1 CTLA-4 and PD-1 PD-1 CTLA-4 or PD-1	R R R R ND R R ND R R ND, R	1,2 1 2 1 4 1 2 2,3 1 2 1,2	R ANR ANR R R R R ANR ANR NYR NYR	NCT04977375 NCT03961971 NCT04225039 NCT04656535 NCT05235737 NCT05106296 NCT04145115 NCT04396860 NCT04323046 NCT05909618 NCT06047379
Cell Therapy	INFα2 αCD3 x αEGRF BATs	ND ND	1,2 1	R ANR	NCT03866109 NCT03344250
Cancer Vaccine	DNA vaccine pp65 and gB Survivin, PD-1 and GM-CSF mutation-derived tumor antigen vaccine RNA-loaded lipid particles Oncolytic HSV TVI-Brain-1 HSPPC-96 Oncolytic HSV Oncolytic HSV Peptide H3K27M	ND R R ND ND R ND ND R R ND	1 1,2 2 1 1 1 2,3 2 1 1 1	R R R ANR R ANR R R R R R	NCT05698199 NCT03382977 NCT04013672 NCT03223103 NCT04573140 NCT02062827 NCT05685004 NCT03650257 NCT03657576 NCT03152318 NCT04808245

1. Target UNK = undisclosed.

2. Disease stage TN, Treatment Naïve; ND, Newly Diagnosed glioblastoma; R, Recurrent glioblastoma; ST, Any solid tumor meeting specific requirements.

3. Status R, Recruiting; ANR, Active not recruiting; NYR, Not yet recruiting.

## 7 Conclusions/Future directions

It is recognized that solid tumor control by the immune system requires a multi-step process involving different immune cells, including DCs and T cells (74). However, recent studies suggest that the unique glioblastoma microenvironment, influenced by its intracranial location, results in a particular deficit in DC number and function (75, 85, 86), T cell sequestration in the bone marrow (80), and the suppressive TME (69) all pose hurdles to be overcome in producing effective immunotherapies. Friebel et al. reported interesting links between the immune cells present in the TME of glioblastoma, in that more T cells were associated with more DCs, and conversely, more TAMs were associated with fewer T cells (9). Lee et al. also concluded that the recruitment of T cells and DCs in combination with other immunotherapies would be required to achieve significant results (150). Likewise, Liu et al. conclude that the future of glioblastoma treatments lies in a multifaceted immune response (129). Eiraku et al. had a similar conclusion that combinations of DCV and CAR-T cells with ICI may provide a better outcome (53).

Immunotherapies aim to enhance or enable the patient’s immune system to detect, target and kill cancer cells while protecting normal cells (104, 151). However, the immune system suppression caused by radiotherapy and chemotherapy has raised questions about the effectiveness of using immunotherapies as a treatment for glioblastoma (152). Many of the suppressive attributes of glioblastoma mentioned above likely also contribute to the limited progress in immune-based treatments for glioblastoma (5). Moreover, although corticosteroids such as dexamethasone are given to glioblastoma patients to reduce inflammation in the brain and relieve symptoms, these agents can reduce immune responses, including a loss of antigen presentation by DCs (88, 153–155). Thus, these standard methods to manage glioblastoma symptoms may further limit the effectiveness of immunotherapies. A careful balance between promoting immune function and limiting inflammation in the brain to prevent unwanted side effects must be considered in designing immunotherapeutic approaches (156). As treatments are further developed, the consideration of engaging and supporting all arms of the immune system while targeting the tumor will ultimately lead to better patient outcomes. However, as seen in **Table 2**, less than 25% of the current glioblastoma trials in **Table 2** involve combined immunotherapies. Highlighting the need for combination therapies in glioblastoma is the remarkable report by Zhu et al. that describes a single glioblastoma patient with a progression-free survival of 69 months following treatment with a personalized DCV combined with depletion of T_reg_ cells, ICI with anti-PD-1, and an immune adjuvant Poly I:C (157).

We propose that the approaches most likely to succeed will find a way to enhance the suboptimal T cell and DC populations present in glioblastoma patients, likely through combination treatments targeting both ends of the DC – T cell axis.

## Author contributions

BG: Conceptualization, Writing – original draft, Writing – review & editing. TG: Conceptualization, Supervision, Writing – review & editing. MB: Supervision, Writing – review & editing. LE: Conceptualization, Supervision, Writing – review & editing.
